# Bone marrow mesenchymal stem cell exosomes suppress phosphate-induced aortic calcification via SIRT6–HMGB1 deacetylation

**DOI:** 10.1186/s13287-021-02307-8

**Published:** 2021-04-13

**Authors:** Wenqian Wei, Xiaodong Guo, Lijie Gu, Jieshuang Jia, Man Yang, Weijie Yuan, Shu Rong

**Affiliations:** 1 Department of Nephrology, Shanghai General Hospital, Shanghai Jiaotong University School of Medicine, No. 100, Haining Rd, Hongkou District, Shanghai, 200080 China; 2 Department of Oncology, Yueyang Hospital of Traditional Chinese and Western Medicine Affiliated to Shanghai University of Traditional Chinese Medicine, Shanghai, 200437 China

**Keywords:** Chronic kidney disease, Vascular calcification, Bone marrow mesenchymal stem cells, Exosomes, SIRT6, HMGB1

## Abstract

**Background:**

Vascular calcification associated with chronic kidney disease (CKD) can increase the risk of mortality. Elevated serum levels of high mobility group box 1 (HMGB1) promotes vascular calcification in CKD via the Wnt/β-catenin pathway. Sirtuin 6 (SIRT6) prevents fibrosis in CKD by blocking the expression of β-catenin target genes through deacetylation. This study aimed to investigate whether the inhibition of vascular calcification by bone marrow mesenchymal stem cell (BMSC)-derived exosomes is related to SIRT6 activity and assess the regulatory relationship between HMGB1 and SIRT6.

**Methods:**

CKD characteristics, osteogenic markers, calcium deposition, and the differential expression of HMGB1 and SIRT6 have been measured in a 5/6 nephrectomized mouse CKD model fed a high-phosphate diet to induce aortic calcification. In vitro assays were also performed to validate the in vivo findings.

**Results:**

High phosphate promotes the translocation of HMGB1 from the nucleus to the cytosol and induces the expression of *Runx2*, osteopontin, and *Msx2*. However, BMSC-derived exosomes were found to alleviate CKD-related fibrosis and the induction of osteogenic genes although less significantly when SIRT6 expression is suppressed. SIRT6 was found to modulate the cytosol translocation of HMGB1 by deacetylation in vascular smooth muscle cells.

**Conclusion:**

Our results indicate that BMSC-derived exosomes inhibit high phosphate-induced aortic calcification and ameliorate renal function via the SIRT6–HMGB1 deacetylation pathway.

## Background

Vascular calcification associated with chronic kidney disease (CKD) is a condition of multifactor etiology that can increase the risk of cardiovascular mortality [1, 2]. Calcification originates in the aortic walls of vascular smooth muscle cells (VSMCs) in response to increasing levels of inorganic phosphate (Pi) [3]. The current management of vascular calcification includes dietary adjustments to limit the intake of phosphates and adjust the levels of Ca, parathyroid hormone, and vitamin D but these strategies do not greatly impact mortality rates [4, 5]. A better understanding of the molecular processes involved in vascular calcification may contribute to improved management strategies.

High mobility group box 1 (HMGB1) is a nuclear protein involved in chromatin remodeling through interactions with transcription factors, histones, and nucleosomes [6]. HMGB1 is thought to play a role in CKD through its association with chronic inflammation [7]. In a previous study, we found that the elevation of HMGB1 is associated with vascular calcification [8]. Moreover, high Pi levels promote the translocation of HMGB1 from the nucleus to the cytosol. We have associated the release of HMGB1 in aortic calcification and kidney damage with the Wnt/β-catenin pathway and the upregulation of bone morphogenetic protein 2 (BMP-2) [8, 9].

The nuclear to cytoplasmic translocation of HMGB1 in CKD is believed to involve acetylation by the Sirtuin class of proteins (SIRT1–7) [10]. Sirtuins control acetylation by either mono-ADP-ribosyltransferase or NAD(+)-dependent histone deacetylase activity [11]. SIRT1 and SIRT6 are known to be associated with vascular system-related diseases [12]. SIRT1 has been implicated as a regulator of calcification because it is associated with β-Catenin/Wnt signaling through elevated Runx2 levels, which subsequently leads to the accumulation of Pi [13, 14]. Less is known about the role played by SIRT6 in vascular calcification although it is known to be involved in the acetylation and translocation of HMGB1 [15]. The deletion of SIRT6 is associated with exacerbated Ang II-induced kidney injury, atherosclerosis, and cholesterol accumulation [16, 17].

In a previous study, exosomes derived from bone marrow mesenchymal stem cells (BMSCs) were found to alleviate the occurrence of high Pi-induced vascular calcification [18]. The nano-size of exosomes and their ability to target specific cells bestows them with several useful molecular functions, including non-invasive therapeutic carriers. The purpose of this study was to investigate whether the inhibition of vascular calcification by BMSC-derived exosomes involves the participation of SIRT6. To investigate the roles of HMGB1 and SIRT6 in aortic calcification, we used a 5/6 nephrectomized mouse CKD model fed a high-Pi diet. To determine whether acetylation is involved in the interactions between SIRT6 and HMGB1, we used co-immunoprecipitation in VSMCs.

## Materials and methods

### Ethics statement

All animal experiments were performed in accordance with the American Animal Protection Legislation. All study protocols were approved by the Institutional Animal Care and Use Committee (IACUC) of Shanghai Jiao Tong University (approval No. A2019-010).

### Isolation and identification of BMSCs

Male C57BL/6J mice aged 4–6 weeks were obtained from the Jiesijie Experimental Animal Company (Shanghai, China). To obtain primary BMSCs, mice were sacrificed by cervical dislocation and bone marrow in the tibias and femurs of the mice was collected in Dulbecco’s modified Eagle medium (DMEM). The resulting suspension was centrifuged at 1000×*g* for 5 min and resuspended in DMEM medium containing 15% fetal bovine serum (FBS). BMSCs cultured for two to three passages before use.

The BMSCs were identified by the surface markers CD29 (ab21845), CD34 (ab23830), CD45 (ab210182), and CD105 (ab184667) in flow cytometry and by osteogenic and adipogenic differentiation assays. Osteogenic differentiation was induced in BMSCs with DMEM containing 10% FBS, 5 μg/mL insulin, 0.1 μM dexamethasone, 0.2 mM vitamin C, and 10 mM β-glycerophosphate. The media was changed every 3 days for 3 weeks. Calcium precipitation and nodules were identified by intense Alizarin Red staining. Adipogenic differentiation was induced with DMEM containing 10% FBS, 10 μg/mL insulin, 1% penicillin-streptomycin, and 2 mM glutamine. The media was changed every 3 days for 3 weeks. Oil droplets were detected with Oil red O staining.

### Preparation and identification of BMSC Exosomes

To prepare exosomes, exosome-depleted media was first prepared by centrifuging DMEM at 100,000×*g* for 16 h. BMSCs were cultured for 48 h in the exosome-depleted media. Conditioned media was collected at 4 °C by differential centrifugation: 300×*g* for 5 min, 2000×*g* for 30 min, and 10,000×*g* for 70 min. The final pellet was washed in phosphate-buffered saline (PBS) and exosomes were isolated by centrifugation at 10,000×*g* for 70 min. Exosome-associated markers (CD9, ab92726; CD63, ab216130; and TSG101, ab245448; 1:2000, Abcam, Cambridge, UK) and western blotting were used for confirmation. Exosomes were resuspended in PBS and stored at − 80 °C.

The size of the isolated exosomes was measured using nanoparticle tracking analysis and ZetaView (Particle Metrix, Munich, Germany). Exosome morphology was determined by transmission electron microscopy (TEM). Exosomes (20 μL) were adsorbed to carbon and counterstained with 2% phosphotungstic acid. After airdrying, images were captured on a JEM-1400 Plus transmission electron microscope (JEOL, Tokyo, Japan).

### Construction of lentivirus vectors encoding SIRT6 shRNA

Lentivirus vectors pGLV3/H1/GFP+Puro (Genechem, Shanghai, China) containing cytomegalovirus (CMV)-mediated green fluorescent protein (GFP) were used in constructions. Murine *SIRT6* was targeted by the following shRNA sequence (SIRT6-shRNA): sense 5′-GCATGTTTCGTATAAGTCCAA-3′ and scramble shRNA sequence (NC-shRNA): 5′-GTGCAATGTTTCGCATGTTTG-3′. The RNA sequences were inserted into the *Bam*H1 and *Eco*R1 restriction sites of the lentiviral vectors and amplified in HEK-293 cells.

### Induction of CKD in the mouse model

CKD was induced by 5/6 nephrectomy in male C57BL/6J mice aged 8–10 weeks as described previously [19]. A standard two-stage surgical ablation procedure was used. Mice were anesthetized with sodium pentobarbital (10 mg/kg) intraperitoneally. First, two thirds of the left kidney was excised followed by the complete removal of the right-hand kidney. The procedure was repeated in sham-operated mice without the removal of kidney tissue. Six mice were used for each experiment. Mice were allowed to recover for 1 week and then placed on either a normal (0.5%) or high-phosphate (1.5%) diet. Two weeks following the 5/6 nephrectomy, mice were administered with either 100 μl *SIRT6* shRNA (*SIRT6*-shRNA) or scramble shRNA (NC-shRNA) lentivirus (HanBio Biotechnology, Shanghai, China) by tail vein injection at a dose of 10^8^ per animal. For exosome treatment, exosomes derived from BMSCs were injected into mice (20 μg/mice) via tail vein, twice a week post-surgery, and the same volume of PBS injected as a control. Blood was collected by cardiac puncture for biochemical tests 24 h before the mice were euthanized (50 mg/kg pentobarbital) and thoracic aortas were removed for the isolation of VSMCs, histochemistry, and the determination of Ca deposition.

### VSMC isolation and treatment

Primary mouse VSMCs were isolated from the thoracic aorta of C57BL/6J mice aged 8 weeks and cultured in DMEMs supplemented with 10% FBS and maintained in 5% CO_2_ at 37 °C. VSMCs were plated at a confluence of 50–70% and used at passages 4 to 6. For high-Pi experiments, VSMCs were treated with 2.5 mM ionic phosphate. For the knockdown of *SIRT6*, VSMCs were transduced with NC-shRNA or *SIRT6-*shRNA lentivirus at a multiplicity of infection of 100 for 48 h. Western blot analysis was performed to assess the effectiveness of the *SIRT6* knockdown.

### PKH26-labeled exosomes and tracking in VSMCs

To track ADSCs-Exo in VSMCs, PKH26 (Sigma-Aldrich, St. Louis, MO, USA) and labeled ADSCs-Exo (PKH26-ADSCs-Exo) were added to VSMCs transfected with NC-shRNA or *SIRT6-*shRNA lentivirus and incubated for 24 h. VSMCs were fixed in paraformaldehyde (4%) and nuclei were counterstained with DAPI. Fluorescence was observed and images were captured using a confocal microscope (Olympus, Tokyo, Japan).

### Exosomes tracking in vivo

PKH26-labeled exosomes were injected into CKD mice (20 μg/mice) via a tail vein, twice a week post-surgery for 12 weeks, and the distribution of exosomes in the kidneys was observed by a fluorescence microscope (Nikon, Tokyo, Japan).

### Blood biochemistry

The serum levels of blood urea nitrogen (BUN), creatinine (Cre), Ca, and Pi were measured using commercially available kits (Nanjing Jiancheng Bioengineering Institute, Nanjing, China). ELISA kits were used to measure the serum levels of FGF23 (R&D Systems, Minneapolis, MN, USA) and HMGB1 (Shino-Test, Kanagawa, Japan) according to the manufacturer’s instructions.

### Masson stain

The kidneys of mice were cut in transverse sections, fixed in 10% formaldehyde, and embedded in paraffin. Deparaffinized sections were cut into 10 μM sections and stained with Masson’s trichrome. Interstitial fibrosis and tubular lumen dilatation were measured with ImagePro software (Media Cybernetics, Rockville, MD, USA).

### Hematoxylin and eosin (HE) stain

The thoracic aortas of mice were fixed in 10% formaldehyde and embedded in paraffin. They were then cut into 4-μm sections and stained with HE. Pathological changes in thoracic aortas tissues were observed under a microscope (Olympus).

### Ca and Pi content in thoracic aorta

To determine levels of Ca and Pi in aortas, the aortas were dried at 55 °C and then weighed. Ca and Pi were extracted with 150 mM HCl overnight at room temperature. The Ca and Pi content in the solution was measured using Colorimetric Assay kits (Wako, Osaka, Japan) according to the manufacturer’s instructions. The Ca and Pi content was determined in relation to the dry tissue weight.

### Alizarin Red staining of thoracic aorta and quantification of VSMCs

Alizarin Red stain (1 g/L) was added to deparaffinized aortic sections and incubated for 5–10 min at room temperature. Sections were washed with deionized water and observed under a microscope (Olympus). To quantify Ca deposition in VSMC cultures, 2% Alizarin Red was added to VSMCs and fixed in 95% alcohol for 5 min at room temperature. Cells were washed with deionized water and observed under a microscope. The Alizarin Red staining was eluted with 10% formic acid and the absorbance was determined at 405 nm on a microplate reader.

### Intracellular calcium content

To measure intracellular Ca content, cells were first decalcified in 0.6 mM HCl at 4 °C for 24 h. Ca released by the cells was measured with a Colorimetric Assay kit (Wako, Osaka, Japan) according to the manufacturer’s instructions. The Ca content was normalized to cell protein content using a BCA assay (Pierce, Waltham, MA, USA).

### Immunofluorescence detection in thoracic aorta

To detect immunofluorescence labeled HMGB1 (red) and SIRT6 (green), tissue sections were first washed with PBS and Triton X-100 for 10 min. Sections were then blocked in 10% nonimmune serum for 1 h at room temperature and incubated in SIRT6 (1:100, 13572-1-AP; ProteinTech Group, Inc., Chicago, IL, USA) and HMGB1 (1:100, 66525-1-lg; ProteinTech Group, Inc.) primary antibodies overnight at 4 °C. Sections were then incubated with secondary antibodies for 2 h and then nuclei were counterstained with DAPI (blue) for 2 min and examined under a TE2000-S fluorescence microscope (Nikon, Tokyo, Japan). All antibodies for specific protein examples have been validated with western blot analysis (Supplementary Fig. 1).

### Western blot analysis

To extract proteins from aortic tissue, aortas were frozen and homogenized in RIPA buffer. They were then centrifuged at 12000×*g* at 4 °C for 15 min. A commercial protein separation kit (Thermo Fisher Scientific, Rockford, IL, USA) was used to separate nuclear and cytoplasmic proteins from frozen aortic tissue. The concentration of protein was measured using a BCA assay kit (Beyotime, Jiangsu, China). Equal amounts of protein were separated by SDS-PAGE and then transferred to PVDF membranes. The membranes were incubated with primary antibodies against osteopontin (1:1000, ab8448), Runx2 (1:1500, ab23981), Msx2 (1:1000, ab227720), β-actin (1:1000, ab8227) (Abcam), HMGB1 (1:1000, 66525-1-lg; ProteinTech), and SIRT6 (1:1000, 13572-1-AP; ProteinTech) overnight at 4 °C and then incubated with goat polyclonal anti-rabbit IgG HRP-conjugated secondary antibodies (1:2000, ab97051, Abcam) at room temperature. Immunoreactive proteins were visualized using an ECL Plus detection system (Amersham Biosciences, Little Chalfont, UK) and band intensity was determined using a gel image analysis system (BioRad, Hercules, CA, USA).

### Immunoprecipitation and detection of acetylation

Protein (200 μg) from tissue homogenates and cell lysates were immunoprecipitated with anti-acetylated-lysine antibody (1:100; #94415; Cell Signaling Technology, Danvers, MA, USA). Immunocomplexes were incubated overnight with 20 μl of protein A/G Plus-agarose beads (Santa Cruz Biotechnology, Inc., Santa Cruz, CA, USA) at 4 °C for 2 h. Finally, the beads were added to 1 ml of wash buffer and centrifuged at 4000×*g* for 1 min. The supernatants were then analyzed by western blotting.

### Statistical analysis

Data are presented as the mean ± SD, with *P <* 0.05 considered significant between groups. All experiments were repeated at least three times. Details of the biological replicates are listed in figure legends. Data are means ± standard deviation (SD) of three independent experiments with three replicates per experiment. GraphPad Prism (GraphPad, San Diego, CA, USA) was used for statistical testing. Samples were compared either via unpaired Student’s *t*-tests or one-way ANOVAs with Bonferroni’s post hoc test as appropriate. *P* < 0.05 was the significance threshold.

## Results

### Identification and isolation of exosomes from BMSCs

**Fig. 1:**
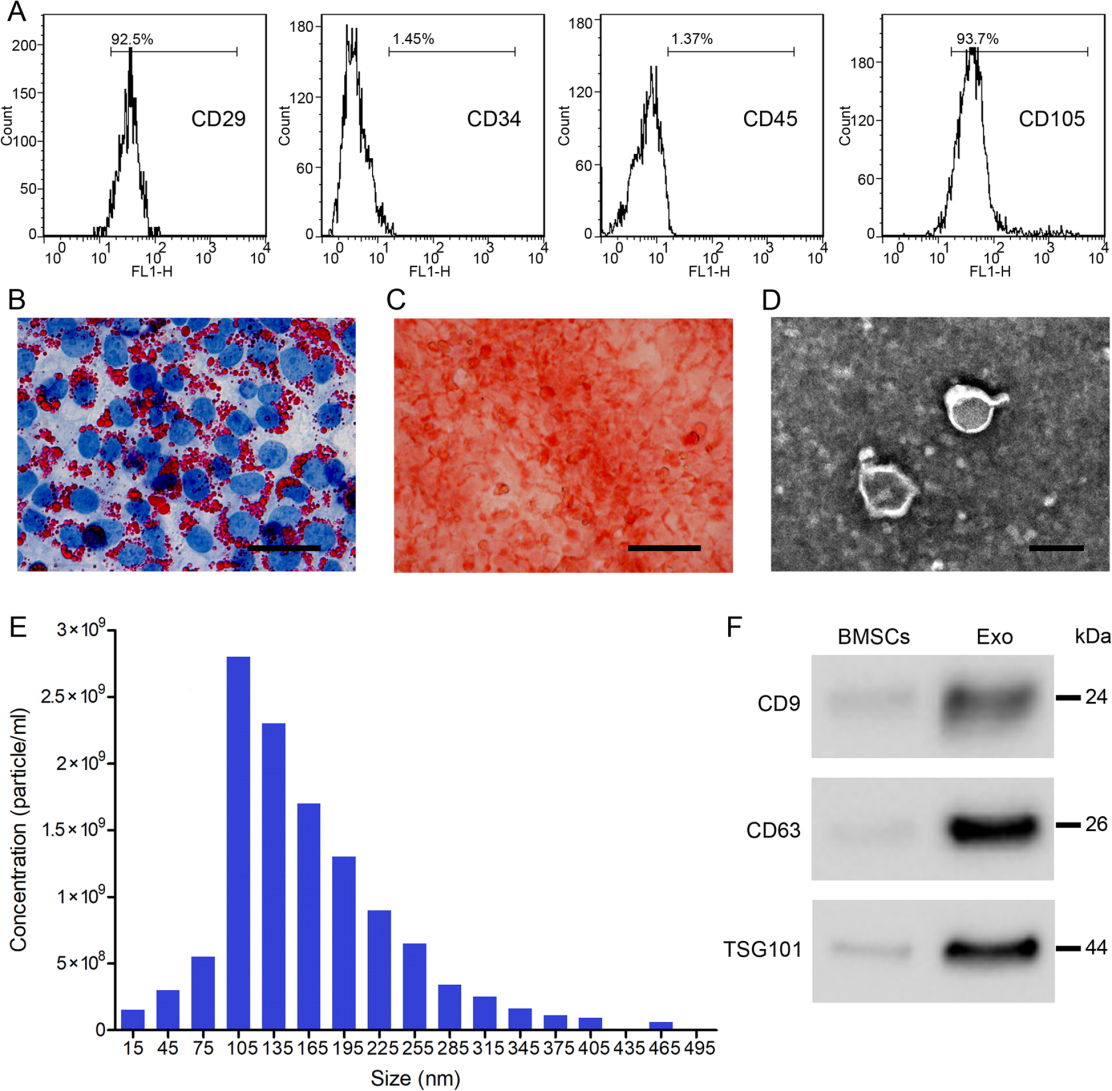
Identification and isolation of bone marrow mesenchymal stem cell (BMSC)-derived exosomes. **a** Flowcytometric analysis indicates that the cells were mesenchymal stem cells since CD29 and CD105 were positive and CD34 and CD45 were negative. **b** Oil red O staining. Scale bar = 50 μm. **c** Alizarin Red staining. Scale bar = 50 μm. **d** The ultrastructure of BMSC-derived exosomes was analyzed by transmission electron microscopy. Scale bar = 100 nm. **e** Nanoparticle tracking analysis of the exosome diameters (nm). **f** Specific exosomal surface markers (CD9, CD63, and TSG101) were detected by western blotting in BMSCs and exosomes

Primary BMSCs were obtained from the tibia and femur of 4–6-week-old male C57BL/6J mice. They were identified as mesenchymal stem cells in flow cytometry by the expression of the stem cell markers CD29 and CD105 and the absence of CD34 and CD45 expression (Fig. 1a). Several BMSC characterization tests were also conducted, including Oil red staining to distinguish adipogenic differentiation through the appearance of cytoplasmic oil droplets (Fig. 1b) and an Alizarin Red test to confirm osteogenic differentiation through the intense staining of calcium (Fig. 1c). The presence of exosomes was confirmed by TEM and nanoparticle tracking analysis was used to measure the quantity and diameter of the isolated exosomes, which were ~ 100 nm and 2.75 × 10^9^ particles/mL, respectively (Fig. 1d, e). Finally, to confirm the isolation of exosomes from BMSCs, we performed western blot analysis with exosomal surface markers (CD9, CD63, and TSG101), which were at a higher level in the isolated exosomes than in BMSCs (Fig. 1f).

### BMSC-derived exosomes alleviate renal fibrosis and inflammation aortic calcification in mouse model of CKD

**Fig. 2:**
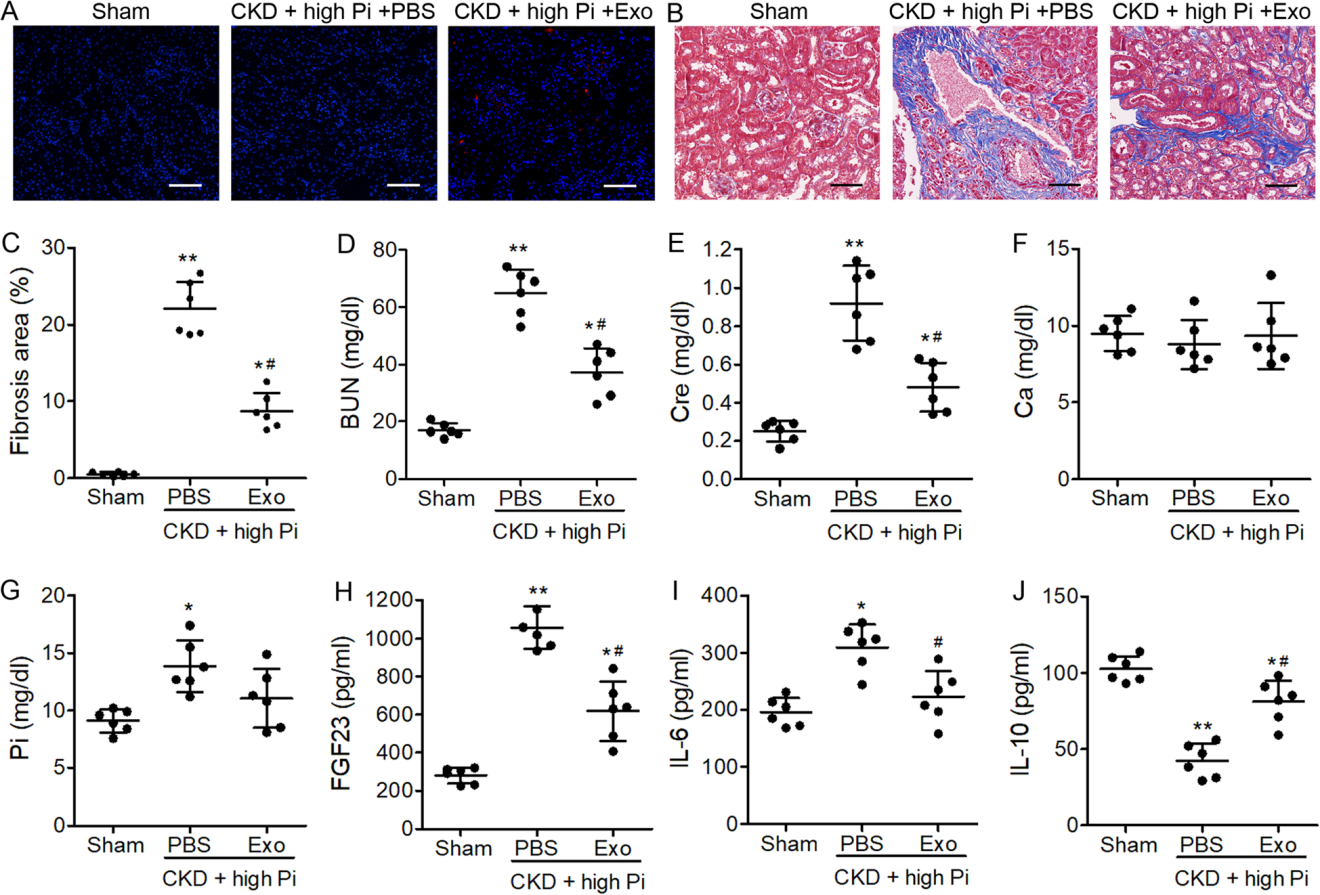
Biochemical analysis of tissue and serum in a murine model of chronic kidney disease (CKD). Sham mice were placed on a normal phosphate (0.5%) diet and CKD mice were placed on high-phosphate (1.5%) diet for 12 weeks with either PBS or exosome treatment. **a** Identification of PKH26-labeled exosomes in the kidney tissues. Scale bar = 100 μm. **b** Masson-trichrome staining of kidneys. Scale bar = 100 μm. **c** Quantitative analysis of the interstitial fibrosis area. Serum concentrations of blood urea nitrogen (BUN, **d**), creatinine (Cre, **e**), calcium (Ca, **f**), phosphorus (Pi, **g**), FGF23 (**h**), IL-6 (**i**), and IL-10 (**j**) were measured by ELISA. *n* = 6 per group. Data are presented as the mean ± SD. *p < 0.05, **p < 0.01, compared with the sham group. ^#^*p* < 0.05 compared with the PBS group

A model of CKD was created in male C57BL/6J mice by performing a 5/6 nephrectomy. The same procedure was performed on sham-operated mice without the removal of kidney tissue. Sham-operated mice were placed on a normal (0.5%) phosphate diet and CKD mice were placed on a high-phosphate (1.5%) diet for 12 weeks and treated with either PBS or BMSC-derived exosomes delivered via tail vein injection. PKH26-labeled exosomes were observed in BMSCs exosomes (CKD+high Pi+Exo) treated group but not in the sham or PBS (CKD+high Pi+PBS) treated groups (Fig. 2a). Severe fibrosis was detected by Masson-trichrome staining of the remaining kidney tissue in mice with CKD (Fig. 2b). However, treatment with BMSC-derived exosomes prevented fibrosis in the kidney tissue of mice with CKD and significantly reduced the fibrotic area (Fig. 2c). Serum concentrations of BUN, creatinine (Cre), and FGF23 were all significantly reduced by treatment with BMSC-derived exosomes. Levels of Ca were similar in sham-operated mice and the CKD models; however, Pi levels were reduced by the exosome treatment but remained significantly higher than in the sham-operated mice (Fig. 2d–h). Finally, protein expression of the pro-inflammatory cytokine IL-6 (Fig. 2i) was significantly decreased, while anti-inflammatory cytokine IL-10 (Fig. 2j) was significantly increased in CKD+high Pi+Exo group compared to the CKD+high Pi+PBS group. Our results demonstrate that kidneys are subjected to damage and are dysfunctional in the CKD model. However, BMSC-derived exosomes can reduce inflammation and inhibit the damage caused by high levels of Pi. Moreover, our results suggest that serum Ca levels remain similar in the sham-operated mice and the CKD models.

### BMSC-derived exosomes alleviate aortic calcification in a mouse model of CKD

**Fig. 3:**
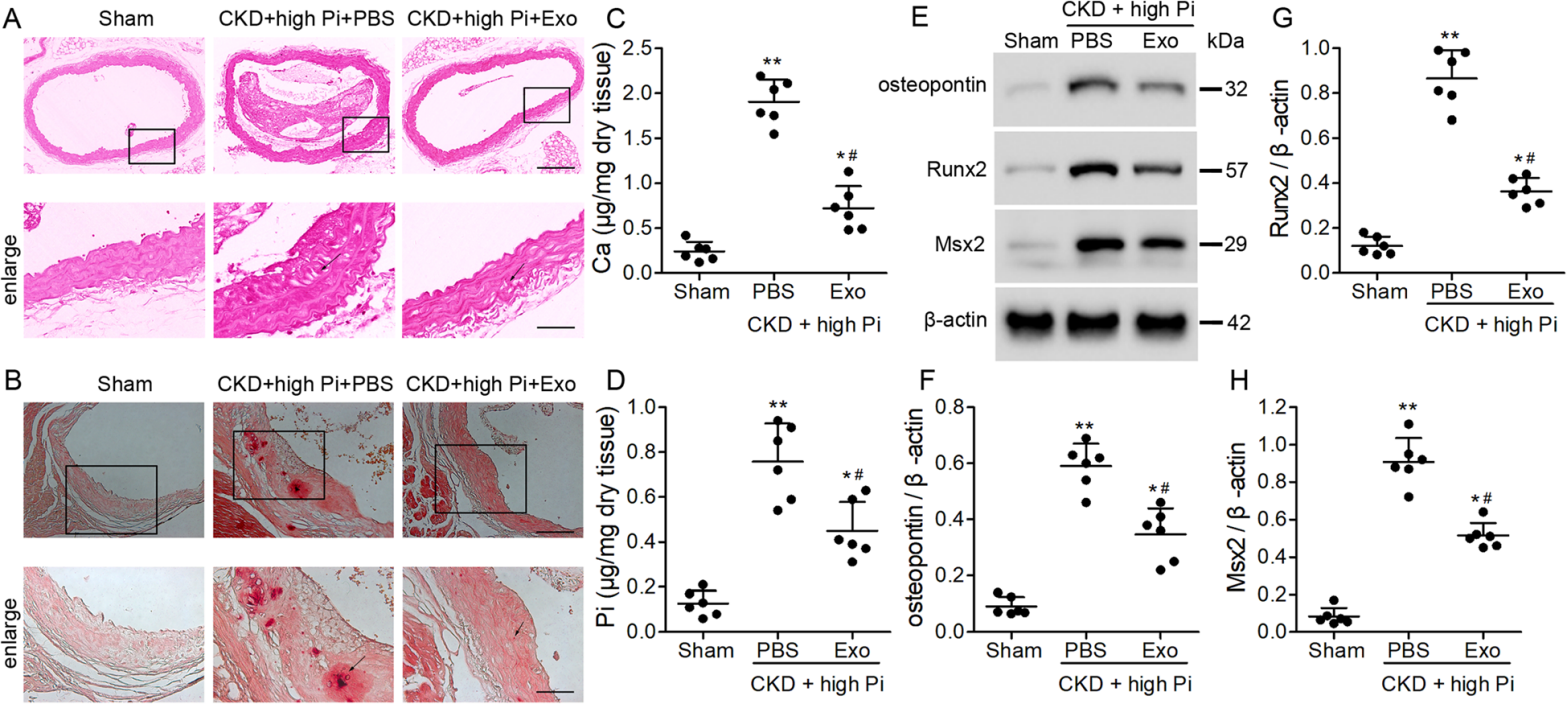
Deposition of Ca in the aorta of a chronic kidney disease (CKD) mouse model. CKD mice were placed on a high-phosphate diet for 12 weeks with either PBS or exosome treatment. **a** Representative micrographs of HE stained sections of the aortas. Scale bar = 200 μm or 50 μm. **b** Representative micrographs of Alizarin Red stained sections of the aortas. Scale bar = 100 μm or 50 μm. **c** Calcium and **d** phosphorus content in the thoracic aortas. **e** Western blot analysis of osteopontin, Runx2, and Msx2 in the aortas of sham-operated and CKD mice. Quantification of osteopontin (**f**) *Runx2* (**g**), and *Msx2* (**h**) expression relative to that of β-actin. *n* = 6 per group. Data are presented as the mean ± SD. *p < 0.05, **p < 0.01, compared with the sham group. ^#^*p* < 0.05 compared with the PBS group

The structure of the aortas was first observed by HE-staining. As showed in Fig. 3a, aortas in the sham group exhibited a compact structure, and the aortas from the CKD+high Pi+PBS group showed a looseness of structure. Treatment with BMSCs exosomes mitigated this structural change. Next, we measured the Ca and Pi content in aortas under the same conditions. In contrast with the levels of Ca found in the serum, levels of Ca in sections of aorta measured by Alizarin Red were significantly higher in the CKD mouse models but treatment with BMSC-derived exosomes reduced the level of Ca in the aorta (Fig. 3b, c). Pi content was also higher in the aorta of the PBS treated mice compared with those treated with exosomes (Fig. 3d). Finally, to determine whether calcification was involved in the deposition of Ca and Pi in the aorta of the CKD mouse model, we assessed the expression of the osteogenic genes, *Runx2*, osteopontin, and *Msx2*, by western blotting (Fig. 3e–h). BMSC-derived exosomes could inhibit the expression of *Runx2*, osteopontin, and *Msx2* but levels were still higher than in sham-operated mice. Overall, these results indicate that CKD and high levels of Pi lead to aortic calcification and that treatment with BMSC-derived exosomes might inhibit aortic calcification through decreasing the expression of osteogenic genes and the deposition of Ca.

### SIRT6 inhibits the cytosol translocation of HMGB1

**Fig. 4:**
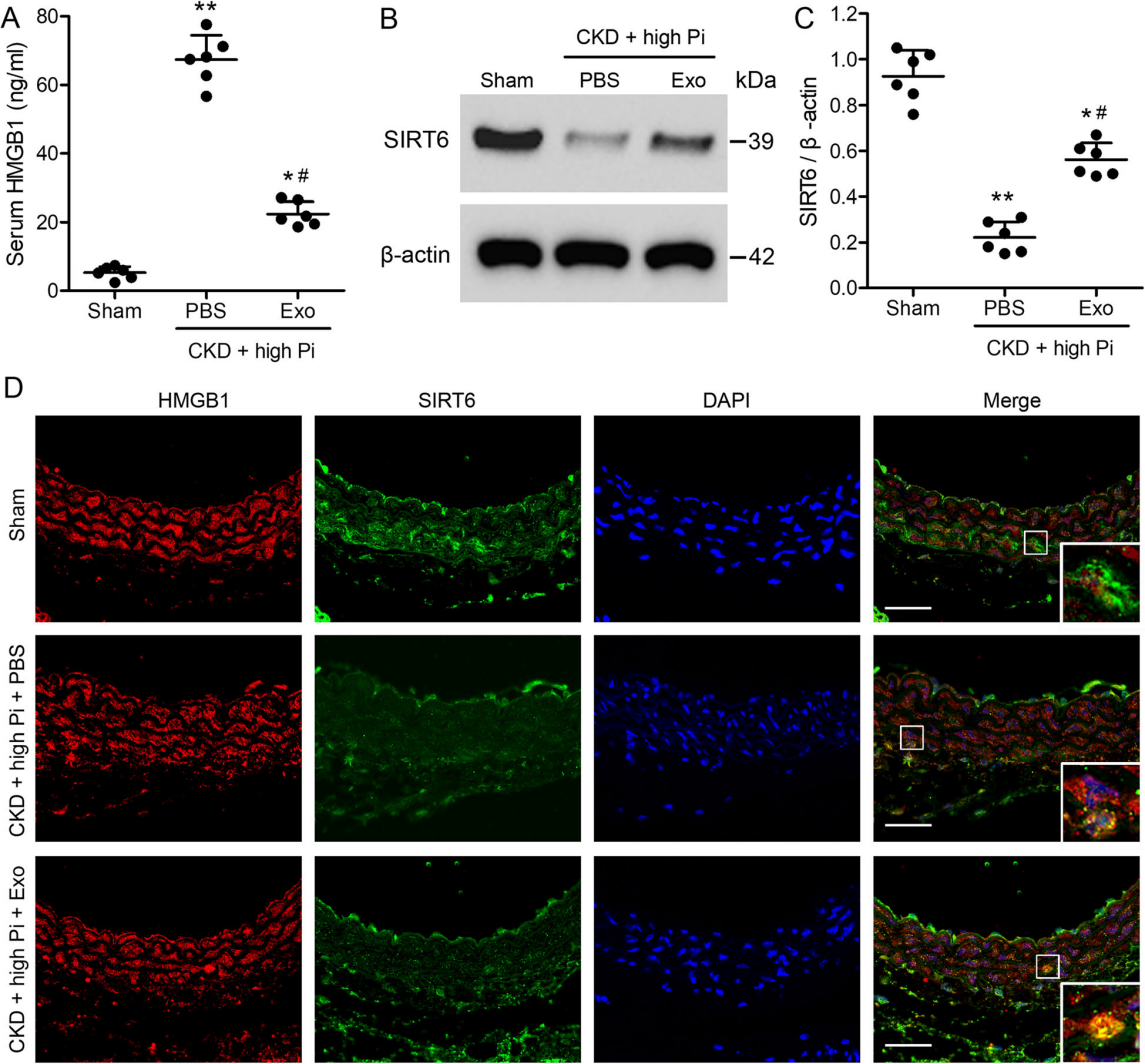
High Pi promotes the translocation of HMGB1 from the nucleus to the cytosol in aortic calcification. CKD mice were placed on a high-phosphate diet for 12 weeks with either PBS or exosome treatment. **a** Serum concentrations of HMGB1 were measured by ELISA. **b** Western blot analysis of SIRT6 in the aortas from sham and CKD mice. **c** Quantification of SIRT6 expression relative to that of β-actin. *n* = 6 per group. Data are presented as the mean ± SD. *p < 0.05, **p < 0.01, compared with sham group. #*p* < 0.05 compared with the PBS group. **d** Immunofluorescence detection of HMGB1 (red) and SIRT6 (green). Nuclei were counterstained with DAPI (blue). Scale bar = 100 μm

To further investigate the inhibition of renal fibrosis and aortic calcification by exosomes, we assessed the characteristics of HMGB1 and SIRT6 in the mouse model of CKD. In the CKD model, serum levels of HMGB1 were significantly higher in the untreated mice than in mice treated with exosomes (Fig. 4a). Levels of HMGB1 were the lowest in the sham-operated mice. In contrast, levels of SIRT6 were highest in the sham-operated mice and the lowest in the untreated mice with CKD whereas levels of SIRT6 were higher in mice treated with exosomes (Fig. 4b, c). This indicated that exosomes treatment could increase SIRT6 levels of mice. Figure 4d demonstrates that high levels of Pi in the aortic tissue of the CKD model promote the translocation of HMGB1 (red) from the nucleus (blue) to the cytosol, whereas increased levels of SIRT6 (green) prevents the cytosol translocation of HMGB1.

### SIRT6 suppression impairs renal function and increases aortic calcification

**Fig. 5:**
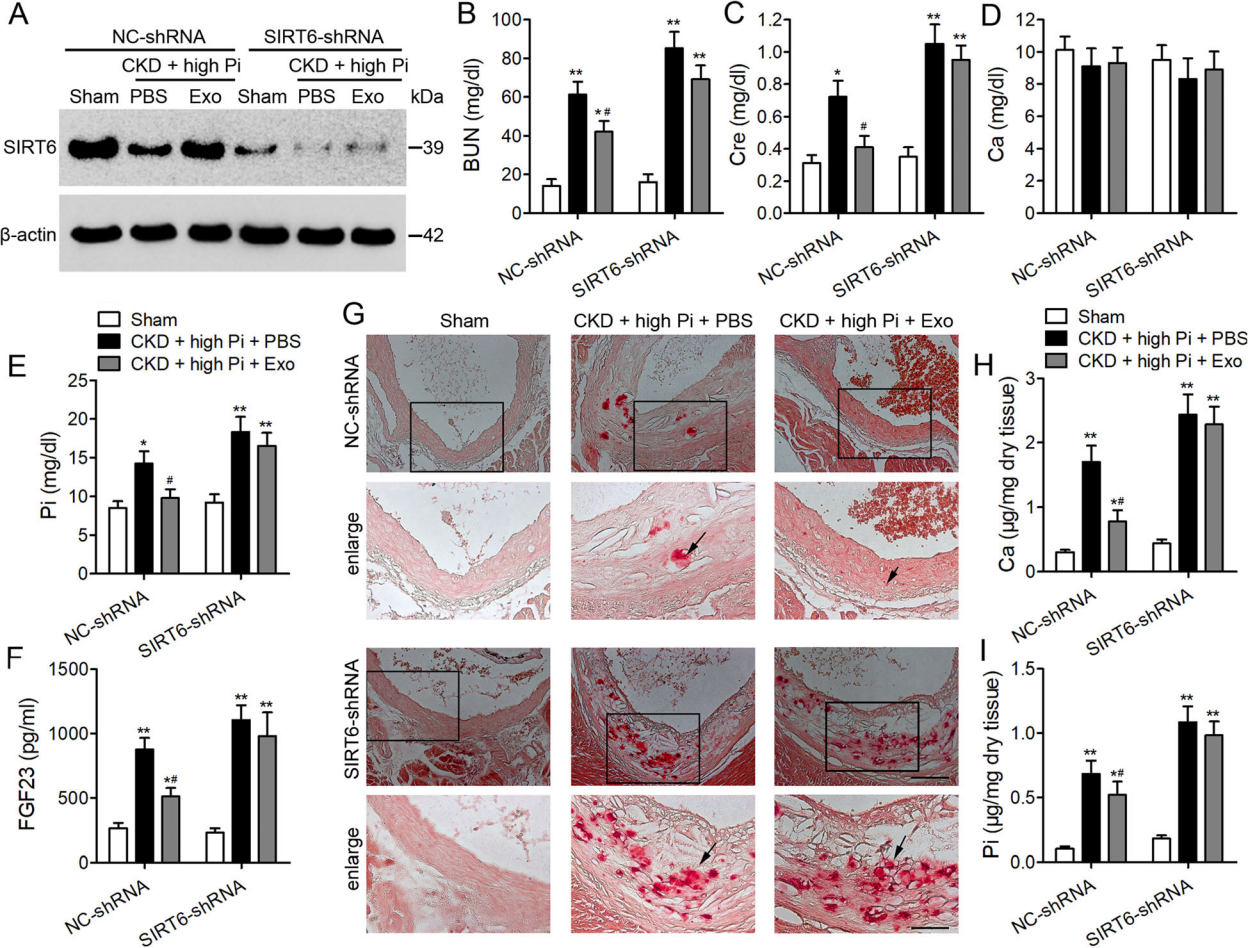
*Sirt6* downregulation impairs renal function and increases aortic calcification in a mouse model of chronic kidney disease (CKD). Mice were administered with scramble shRNA (NC-shRNA) or *Sirt6* shRNA (*Sirt6*-shRNA) lentivirus via tail vein injection. CKD was induced by 5/6 nephrectomy. Sham-operated control mice were subjected to a similar procedure without the removal of kidney tissue. Sham mice were placed on a normal phosphate (0.5%) diet and CKD mice were placed on a high-phosphate (1.5%) diet for 12 weeks with either PBS or exosome treatment. **a** Western blot analysis of SIRT6 in the aortas from sham and CKD mice. Serum concentrations of BUN (**b**), Cre (**c**), Ca (**d**), Pi (**e**), and FGF23 (**f**) were measured by ELISA. *n* = 6 per group. Data are presented as the mean ± SD. *p < 0.05, **p < 0.01, compared with the sham group. ^#^*p* < 0.05 compared with the PBS group in NC-shRNA or *SIRT6*-shRNA lentivirus injection mice. **g** Representative micrographs of Alizarin Red stained sections of the aortas. Scale bar = 100 μm or 50 μm. **h** Calcium and **i** phosphorus content in thoracic aortas

**Fig. 6:**
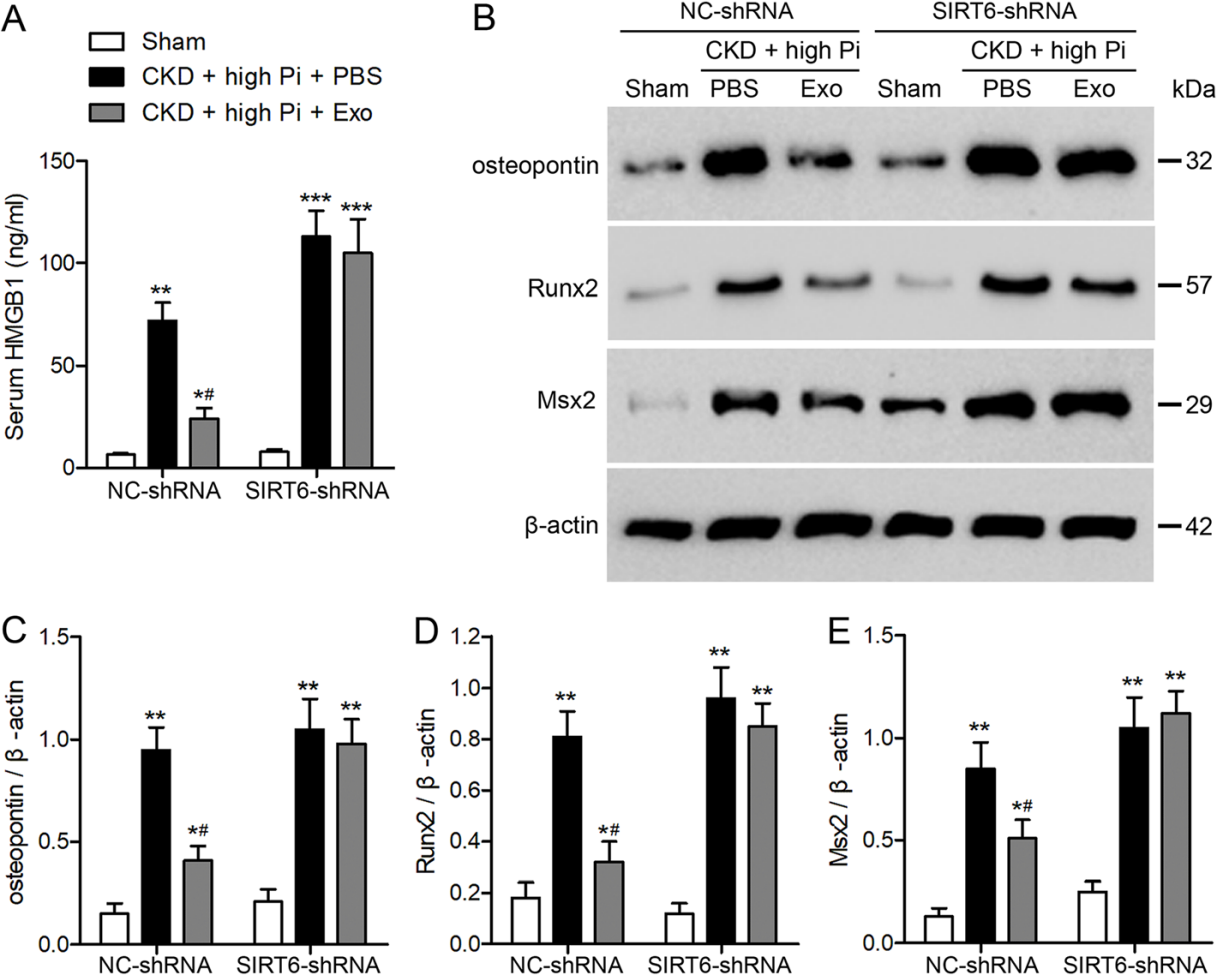
Expression of genes involved in osteogenesis increases when *SIRT6* is downregulated. Chronic kidney disease (CKD) of NC-shRNA or *SIRT6*-shRNA lentivirus injection mice were placed on a high-phosphate diet for 12 weeks with either PBS or exosome treatment. **a** Serum concentrations of HMGB1 were measured by ELISA. **b** Western blot analysis of osteopontin, *Runx2*, and *Msx2* in the aortas from sham-operated and CKD mice. Quantification of osteopontin (**c**), *Runx2* (**d**), and *Msx2* (**e**) expression relative to that of β-actin. *n* = 6 per group. Data are presented as the mean ± SD. *p < 0.05, **p < 0.01, ***p < 0.001, compared with the sham-operated group. ^#^*p* < 0.05 compared with the PBS group in NC-shRNA or *Sirt6*-shRNA lentivirus injected mice

To further understand the involvement of SIRT6 in aortic calcification, we downregulated SIRT6 in vivo by injecting SIRT6-shRNA into the tail vein of the CKD mouse model. Western blotting confirmed a reduction in SIRT6 expression in aortic tissue (Fig. 5a). Serum concentrations of BUN, creatinine, Ca, Pi, and FGF23 indicated that downregulating the expression of SIRT6 resulted in reduced kidney function, however levels of Ca in the serum were not significantly altered (Fig. 5b–f). In contrast, Alizarin Red staining indicated a significantly higher level of Ca deposited in aortic tissue when SIRT6 is downregulated accompanied by higher levels of Pi (Fig. 5g–i). Serum levels of HMGB1 are also elevated when SIRT6 is downregulated (Fig. 6a) with an increased expression of genes involved in osteogenesis (Fig. 6b–e). In particular, the expression of Msx2 is increased when SIRT6 is downregulated (Fig. 6e). These findings demonstrate that SIRT6 plays an important role in enhancing kidney function and preventing aortic calcification in the mouse model of CKD.

### Cytosol translocation of HMGB1 involves SIRT6 deacetylation

**Fig. 7:**
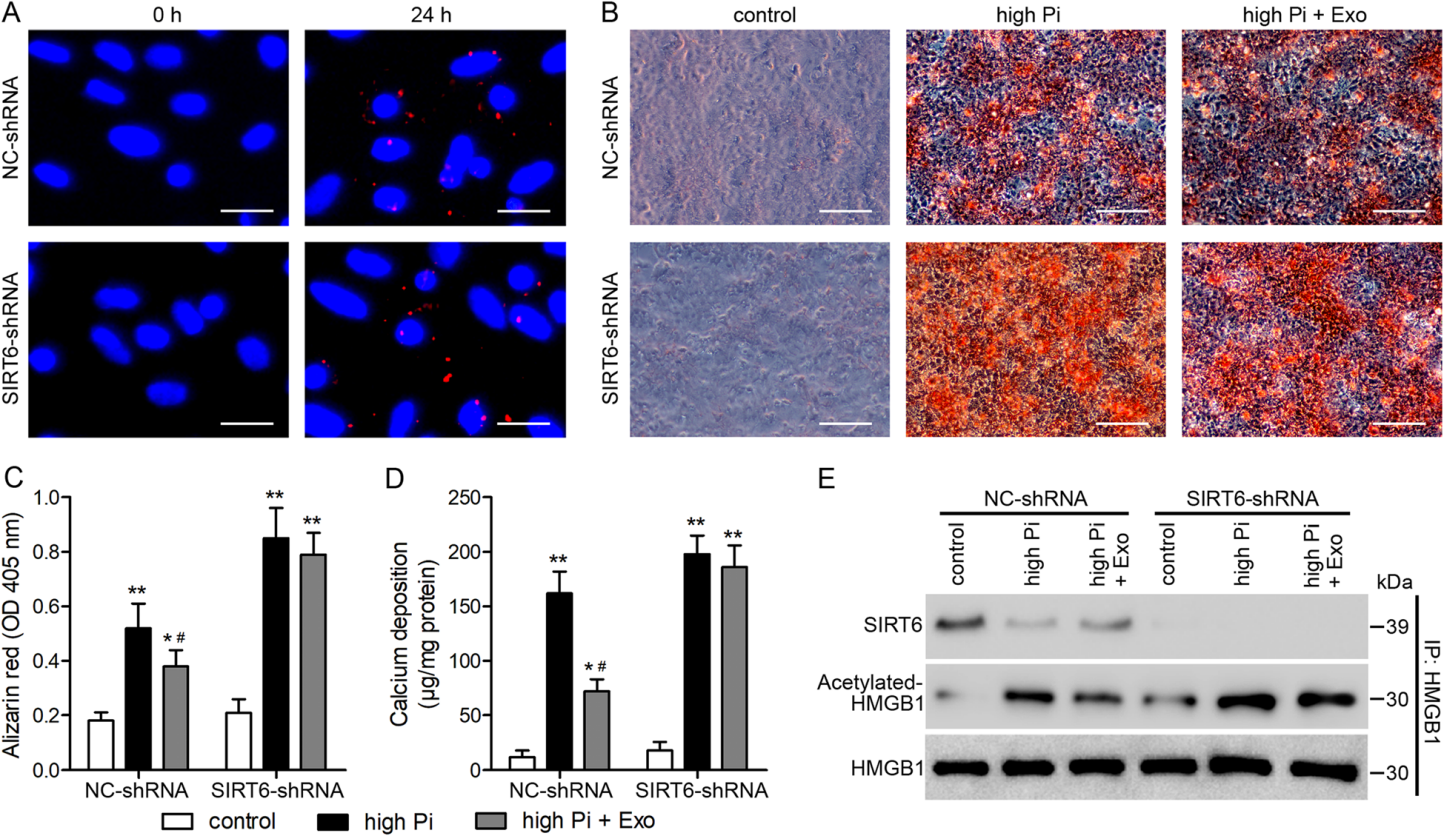
Cytosol translocation of HMGB1 involves deacetylation by SIRT6. **a** Localization of PKH26-labeled bone marrow mesenchymal stem cell (BMSC)-derived exosomes (red) in vascular smooth muscle cells (VSMCs) transfected with NC-shRNA or *SIRT6*-shRNA lentivirus visualized by confocal microscopy at 0 and 24 h post-BMSC exosome incubation. Scale bars = 25 μm. VSMCs transfected with NC-shRNA or *SIRT6*-shRNA lentivirus were treated with high Pi (2.5 mM) for 14 days. **b** Alizarin red staining. Scale bars = 100 μm. **c** Quantification of Alizarin Red staining. **d** Calcium contents measured and normalized by the protein content of cell lysates. *n* = 3 technical replicates. Data are presented as the mean ± SD. *p < 0.05, **p < 0.01, compared with control group. ^#^*p* < 0.05 compared with the high Pi-treated VSMCs transfected with NC-shRNA or *SIRT6*-shRNA lentivirus. **e** Whole-cell lysates were immunoprecipitated with an anti-HMGB1 antibody to determine the interaction with SIRT6. Acetylated HMGB1 levels were also measured

To further understand the interaction between HMGB1 and SIRT6 we conducted in vitro experiments in VSMCs. To determine whether the exosomes could be taken up by different transfected VSMCs, PKH26-labeled BMSC exosomes were incubated with VSMCs transfected with negative control (NC)-small hairpin RNA (shRNA) or SIRT6-shRNA for 24 h, BMSC exosomes were found both in NC-shRNA and SIRT6-shRNA transfected VSMCs (Fig. 7a). Alizarin Red staining indicated the highest levels of Ca in VSMCs treated with Pi without exosomes (Fig. 7b). Exosomes reduced the intensity of Alizarin Red staining and the level of Ca but when SIRT6 was downregulated, levels were similar to VSMCs without exosomes, which indicates that SIRT6 is mainly located in exosomes (Fig. 7c, d). Under control conditions, SIRT6 was co-immunoprecipitated with anti-HMGB1 in whole-cell lysates (Fig. 7e). However, when VSMCs were treated with extracellular Pi, the levels of SIRT6 decreased with a corresponding increase in the levels of acetylated HMGB1, which suggests that the interaction between SIRT6 and HMGB1 involves deacetylation. Moreover, although the levels of SIRT6 were modulated by Pi concentration the levels of HMGB1 remained relatively constant. These results indicate that SIRT6 modulates the cytosol translocation of HMGB1 by deacetylation.

## Discussion

In our study, we demonstrate that the delivery of BMSC-derived exosomes inhibits phosphate-induced aortic calcification and reduced renal fibrosis in a mouse model of CKD. Urea levels of BUN, Cre, and FGF23 were all reduced in the CKD model after the application of exosomes, which indicates improved renal function. This supports similar results found in a recent study, BMSC-derived exosomes were found to reduce the level of alkaline phosphatase (AKP) activity and intracellular Ca thereby inhibiting vascular calcification [18]. The study also found that BMSC-derived exosomes influenced the expression of several miRNAs including those involved in the regulation of Wnt, mTOR, and MAPK signaling, which are associated with vascular calcification [20, 21]. In a previous study, we found that HMGB1 is upregulated during vascular calcification via the Wnt/β-catenin pathway and that high Pi promoted the cytosol translocation of HMGB1 [8], and vascular calcification was attenuated by lowering HMGB1 levels [8, 22]. The binding of HMGB1 to cell surface receptors such as the receptor for advanced glycation end-products (RAGE) and Toll-like receptors (TLRs) is thought to activate Wnt/β-catenin signaling, which in turn activates nuclear factor (NF)-κB to promote a pro-inflammatory response and increased fibrosis [23, 24]. In addition, a recent study discovered that conditioned media from BMSCs could inhibit vascular calcification by blocking the BMP2–Smad1/5/8 signaling pathway [25]. In the study, the BMSC conditioned medium decreased the Ca content of VSMCs and reduced ALP activity and the expression of BMP-2, Runx2, Msx2, and osteocalcin by suppressing the phosphorylation of Smad1/5/8.

In the present study, we found that although serum levels of BUN, Cre, and FGF23 were significantly altered by treatment with BMSC-derived exosomes levels of Ca remained relatively unchanged. However, a significantly higher amount of Ca was deposited in the aorta tissue of the CKD mouse model and high levels of Pi seemed to increase this calcification. A study of vascular calcification in patients with hypoparathyroidism identified that patients at greater risk of developing coronary artery calcification had lower serum Ca levels [26]. However, the exact mechanisms involved are unclear and warrant further investigation.

The expression of the osteogenesis genes Runx2, osteopontin, and Msx2 was also increased in the CKD model but decreased with the addition of BMSC-derived exosomes. Calcium phosphate deposition was once thought to be an entirely passive process; however, it was discovered that osteogenesis was induced during the process [27]. More recently, it has been identified as an entirely active process involving bone development that occurs naturally through aging but is accelerated by underlying health conditions such as CKD [28]. This theory is confirmed in the present study by the upregulation of osteogenic-related genes in the CKD model. An early study found that vesicles in VSMCs exposed to high levels of Ca and Pi contain preformed calcium phosphate, which promotes calcification, whereas serum contains mineralization inhibitors, and the dysfunction of these inhibitors leads to an increase in vascular calcification [29].

After establishing that BMSC-derived exosomes could alleviate the effects of CKD in the mouse model, we next investigated whether this involved the participation of exosomal SIRT6. We found that serum levels of HMGB1 were significantly higher in the untreated mouse CKD model compared to the mouse model treated with exosomes. However, in the presence of exosomes levels of SIRT6 were higher and could prevent the translocation of HMGB1 from the nucleus to the cytosol. Moreover, when SIRT6 expression was downregulated by RNA interference, the exosomes lost their ability to ameliorate the characteristics of CKD.

We used VSMCs to investigate whether the interaction between exosomal SIRT6 and HMGB1 could involve deacetylation. We found that high Pi levels increased the expression of SIRT6 and that SIRT6 modulates the cytosol translocation of HMGB1 by deacetylation. Once in the cytosol, HMGB1 is involved in several cellular processes including autophagy, apoptosis, and the induction of inflammatory factors through the activation of Wnt/β-catenin signaling, which subsequently activates the NF-κB pathway [8, 30].

Recently, SIRT6 has been associated with the prevention of fibrosis in CKD by blocking the expression of β-catenin target genes through deacetylation [31]. The suppression of SIRT1 is known to increase the accumulation of sodium-dependent phosphate co-transporters [32], which in turn leads to a higher level of Pi and the subsequent expression of osteogenic genes and the activation of Wnt signaling. It has been proposed that SIRT6 acts synergistically with SIRT1 because they both translocate to the cytoplasm from the nucleus and are thought to act in similar pathways [12]. In our study, the results show that BMSC-derived exosomes inhibit high phosphate-induced aortic calcification by decreasing the level of HMGB1 via the SIRT6–HMGB1 deacetylation pathway.

## Conclusion

In conclusion, we confirm that the elevation of HMGB1 is associated with high levels of Pi and vascular calcification in CKD. We further established that exosomal SIRT6 was involved in the suppression of osteogenic genes and kidney fibrosis by preventing the cytoplasmic translocation of nuclear HMGB1 by deacetylation. The results of the present study demonstrate that BMSC-derived exosomes can inhibit high phosphate-induced aortic calcification and ameliorate renal and vascular function via the SIRT6–HMGB1 deacetylation pathway. However, the composition of exosomes is very complex, and other mechanisms by which exosomes inhibit vascular calcification need to be studied further.

## Supplementary Information

**Additional file 1.**

## Data Availability

The datasets generated/analyzed during the current study are available.

## Abbreviations

AKP: Alkaline phosphatase
BMP-2: Bone morphogenetic protein 2
BMSCs: Bone marrow mesenchymal stem cells
BUN: Blood urea nitrogen
CKD: Chronic kidney disease
Cre: Creatinine
DMEM: Dulbecco’s modified Eagle medium
FBS: Fetal bovine serum
HMGB1: High mobility group box 1
TLRs: Toll-like receptors
PBS: Phosphate-buffered saline
Pi: Inorganic phosphate
RAGE: Advanced glycation end-products
SD: Standard deviation
VSMCs: Vascular smooth muscle cells
