# Supplementary figures and images for: Bone marrow mesenchymal stem cell exosomes suppress phosphate-induced aortic calcification via SIRT6–HMGB1 deacetylation
Source: Stem Cell Res Ther. 2021 Apr 13;12:235. doi: 10.1186/s13287-021-02307-8 (PMC8042866; doi:10.1186/s13287-021-02307-8)

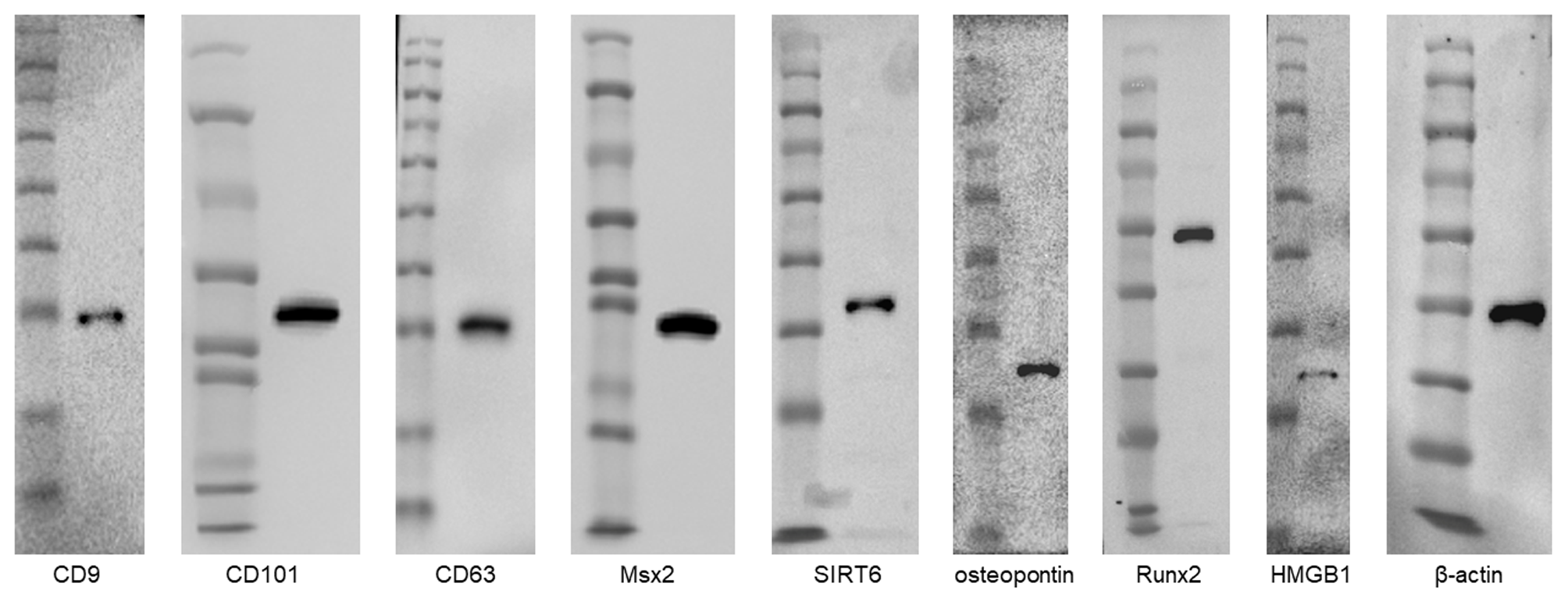

Supplement: Supplementary file 1 — Additional file 1. [file 13287_2021_2307_MOESM1_ESM.tif]
